# Long-term follow-up of patients treated with radiotherapy alone for early-stage histologically aggressive non-Hodgkin's lymphoma

**DOI:** 10.1038/sj.bjc.6601675

**Published:** 2004-02-24

**Authors:** J Spicer, P Smith, K Maclennan, P Hoskin, B Hancock, D Linch, R Pettengell

**Affiliations:** 1 Department of Oncology, St George's Hospital Medical School, Cranmer Terrace, London SW17 ORE, UK; 2 British National Lymphoma Investigation (BNLI), Cancer Research UK and UCL Cancer Trials Centre, 222 Euston Road, London NW1 2DA, UK; 3 Mount Vernon Hospital, Rickmansworth Road, Northwood, Middlesex HA6 2RN, UK; 4 University of Sheffield, Weston Park Hospital, Whitham Road, Sheffield S10 2SJ, UK; 5 Department of Haematology, St George's Hospital Medical School, Cranmer Terrace, London SW17 ORE, UK

**Keywords:** aggressive lymphoma, non-Hodgkin's lymphoma, old age, radiotherapy

## Abstract

Historically localised aggressive non-Hodgkin's lymphoma (NHL) has been treated with involved field radiotherapy (RT), chemotherapy, or a combination of both modalities. The current weight of evidence supports a preference for combined modality treatment (CMT). Increased patient age at diagnosis is well recognised as a poor prognostic indicator in NHL, but despite this some perceive CMT as too toxic for use in the elderly. As a result, some older patients continue to be offered RT alone. Here, we present long-term follow-up of 377 adults of all ages treated with RT alone for early-stage diffuse large-cell lymphoma on British National Lymphoma Investigation trials between 1974 and 1997. 10-year cause-specific survival in patients older than 60 years was poor and significantly inferior to that in younger patients (47 and 75% respectively; *P*<0.001). There is growing evidence that short-course chemotherapy, with or without RT, is superior to RT alone in early-stage aggressive NHL, in elderly as well as in younger patients. Increased age alone should not exclude patients from systemic treatment for early-stage aggressive NHL.

Early stage at diagnosis is a significant predictor of improved outcome in aggressive non-Hodgkin's lymphoma (International Non-Hodgkin's Lymphoma Prognostic Factors Project, 1993). Until relatively recently treatment with involved field radiotherapy (RT) alone, omitting systemic therapy, was an accepted treatment option in patients with localised disease (Ann Arbor stages I and II). However, the combination of chemotherapy with RT has now become more widely used in early-stage disease (Tondini *et al*, 1993; Glick *et al*, 1995; Shenkier *et al*, 2002), and several investigators have commented that this has coincided with improvement in survival over the past two decades (Mauch *et al*, 1985; van der Maazen *et al*, 1998; Tsang and Gospodarowicz, 2001). Two randomised comparisons of combined modality therapy (CMT) against RT alone have confirmed the superiority of CMT (Monfardini *et al*, 1980; Nissen *et al*, 1983). Furthermore, CMT using RT and short-course chemotherapy improved survival compared to full-course chemotherapy alone (Miller *et al*, 1998), although longer follow-up has demonstrated a convergence of both failure-free and overall survival (OS) after 8 years (Miller *et al*, 2000). Recently, the GELA group have demonstrated superior survival with an intensified chemotherapy regimen alone compared with CMT in low-risk patients (Reyes *et al*, 2002).

It has been suggested that the toxicity of chemotherapy is disproportionate to any improvement in outcome in elderly patients, and that short-course RT alone is an adequate treatment for this group. There is some evidence that elderly NHL patients experience more morbidity and mortality from chemotherapy (Armitage and Potter, 1984), but performance status (PS) rather than chronological age seems the most significant predictor of treatment-related death (Gomez *et al*, 1998). Furthermore, the elderly may have more to gain from CMT; increased age is an independent adverse prognostic indicator in aggressive lymphoma (Sutcliffe *et al*, 1985; Kaminski *et al*, 1986; International Non-Hodgkin's Lymphoma Prognostic Factors Project, 1993; Vaughan Hudson *et al*, 1994; Wylie *et al*, 1998; Oguchi *et al*, 2000). There is evidence that the omission of chemotherapy in this age group results in poor outcome. 5-year progression-free survival for a group older than 70 years treated with RT alone was only 31% (Wylie *et al*, 1998), compared to 70% in patients over 65 years given RT plus short-course chemotherapy (Oguchi *et al*, 1997).

Data for stage I aggressive lymphoma patients from the British National Lymphoma Investigation (BNLI) have previously shown 10-year disease-free survival (DFS) and OS rates of 45 and 61%, respectively (Vaughan Hudson *et al*, 1994), comparable to other published series (Jones *et al*, 1973; Chen *et al*, 1979; Reddy *et al*, 1989). Here, we present a retrospective analysis of 377 patients diagnosed with stage IA or IIA diffuse large-cell lymphoma and registered with the BNLI between 1974 and 1997. All patients were treated at presentation with RT alone.

## PATIENTS AND METHODS

### Patients

We have performed a retrospective analysis of 377 adult patients entered into BNLI studies in the period 1974–1997. In all cases, the initial treatment was involved field RT to a dose of 35–40 Gy. The analysis included only patients with a histological diagnosis of diffuse large-cell lymphoma carried out at the treating centre. Central review of histology was performed in all cases. Measurements of serum lactate dehydrogenase (LDH) were not recorded for the early part of this series and so it was not possible to identify accurately patients with raised LDH. Performance status data were not sufficiently complete for analysis. A number of parameters that were recorded, including ESR, were retrospectively analysed along with age and stage as a surrogate for the modified International Prognostic Index (IPI) (Miller *et al*, 1998). All patients underwent a bone marrow examination that excluded lymphomatous infiltration. Primary gut lymphomas were excluded as these may be a distinct biological entity requiring other treatment modalities (Isaacson and Wright, 1984; Armitage *et al*, 2001).

### Statistical analysis

Complete response (CR) was defined as the disappearance of all signs, symptoms and abnormal investigations due to disease for 3 months after completion of initial therapy. Disease-free survival was measured from the start of treatment to the date of recurrence. Recurrence was defined as the development of tumour following a CR to initial treatment. Overall survival included deaths from all causes, whereas cause-specific survival (CSS) was derived by censoring deaths not due to lymphoma or its treatment. Survival was calculated by the life-table method, survival curves were calculated according to the Kaplan–Meier method (Kaplan and Meier, 1958) and statistical comparison of curves was performed using the log-rank test (Peto *et al*, 1971). Multivariate analysis was performed using Cox's proportional-hazards model (Cox, 1972), the variables included in the analysis being age, stage (Ann-Arbor I or II), PS, sex, disease site, ESR, haemoglobin concentration and serum albumin.

## RESULTS

### Patient characteristics

In all, 424 patients were treated with RT alone for early-stage diffuse large-cell lymphoma and were registered with the BNLI during the accrual period. 47 individuals were excluded at registration because of previous malignancy, incomplete staging or lack of other baseline data, thus this analysis was confined to the remaining 377 patients. Median follow-up to date for this group is 12.7 years. There were 202 males and 175 females. Median age was 59 years (range 17–89 years). Patient characteristics are outlined in Table 1

**Table 1:** Patient characteristics

No. of patients	377
Median age (range) (years)	59 (17–89)
Sex ratio (male : female)	1.15 : 1
Stage IA	292 (77%)
Stage IIA	85 (23%)

*Disease site*	
Nodal disease only	189 (50%)
Extranodal only	136 (36%)
Nodal and extranodal	49 (13%)
Unknown	3 (1%)

.

### Results of treatment

A total of 294 patients (78%) achieved a complete remission. The complete remission rate for patients aged less than 60 years was 80%, and for older patients 77%. The CR rates for stages I and II disease were 85 and 58%, respectively. Of those patients achieving complete remission, 57% remained disease free at 10 years. The percentage of all treated patients remaining disease free at 10 years was 44%. The great majority of relapses occurred within the first 5 years following initial treatment. The OS at 10 years was 51%, rising to 63% when patients dying from causes other than lymphoma or its treatment were excluded.

### Subgroup analysis

**Figure 1:**
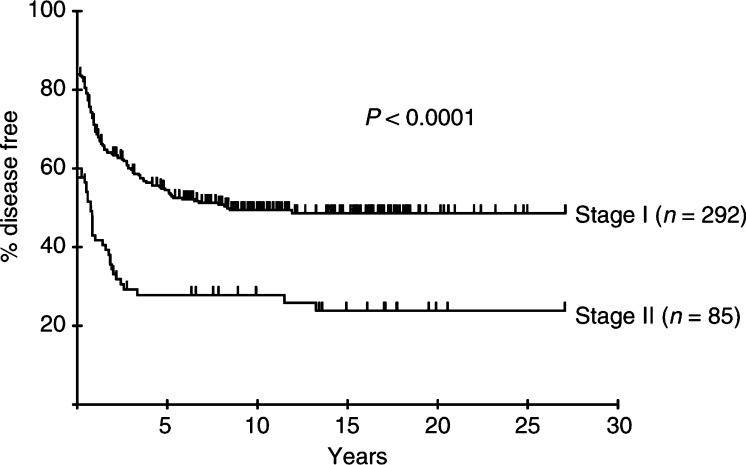
Percentage of patients with early aggressive NHL remaining disease free after initial RT according to stage.

Univariate analysis of DFS identified age (⩾60 years), stage (II *vs* I), ESR (⩾40 mm h^−1^) and serum albumin (<40 g l^−1^), but not haemoglobin, as factors related to inferior DFS. Lactate dehydrogenase was not recorded for many of the patients in this series. Multivariate Cox's regression analysis of DFS identified age (relative risk (RR) 1.45, 95% confidence interval 1.07–2.00), stage (RR 2.19, 95% CI 1.57–3.04) and ESR (RR 2.15, 95% CI 1.30–3.55) as significant risk factors related to relapse. The DFS at 10 years for the patients aged 60 years and over was 35%, compared to 52% for the younger group. The percentage of patients in each stage and age group remaining disease free following initial RT is shown in Figures 1

and 2

**Figure 2:**
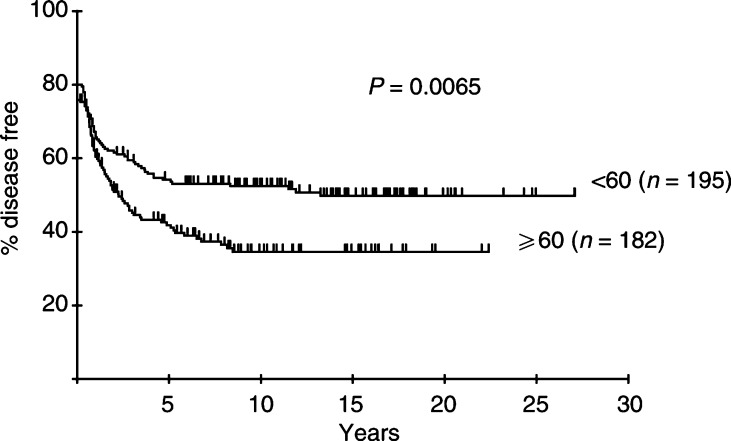
Percentage of patients with early aggressive NHL remaining disease free after initial RT according to age.

.

**Figure 3:**
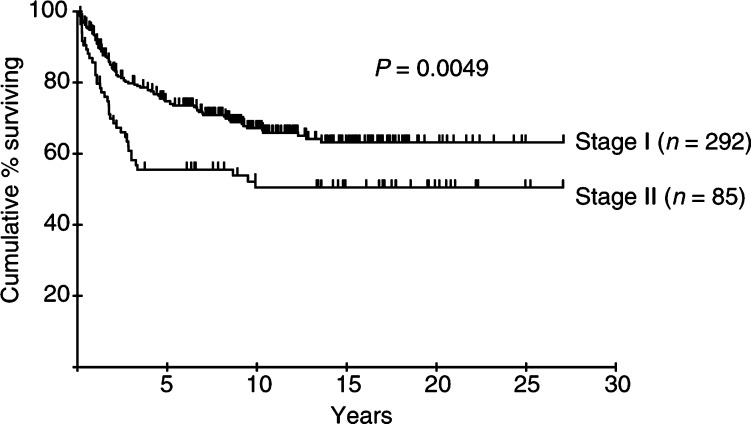
Cause-specific survival according to stage of patients with early aggressive NHL.

Age, stage, ESR and serum albumin were also identified as risk factors related to CSS. Disease site (nodal *vs* extranodal) did not predict survival in this series. Age (RR 2.92, 95% CI 1.94–4.39), stage (RR 1.71, 95% CI 1.11–2.65) and serum albumin (RR 1.55, 95% CI 1.01–2.39) remained significant following multivariate analysis. The survival curves for patients in each stage and age group are shown in Figures 3

and 4

**Figure 4:**
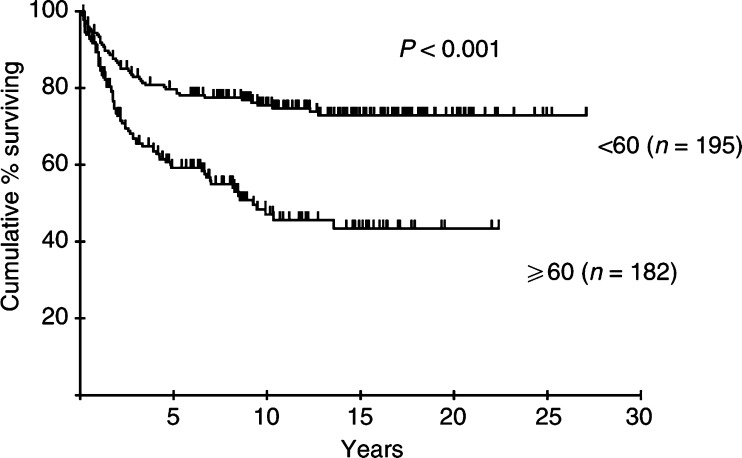
Cause-specific survival according to age of patients with early aggressive NHL.

**Figure 5:**
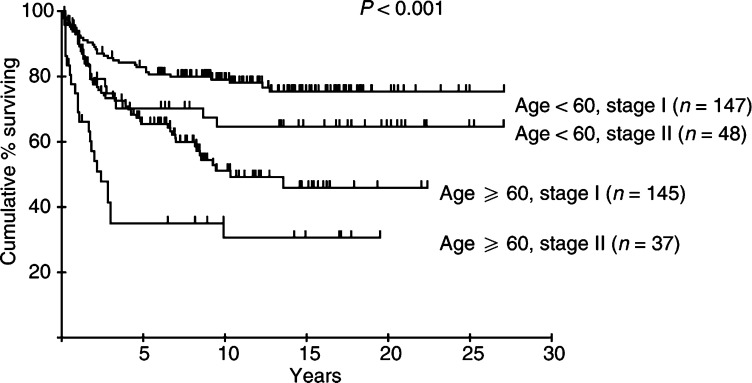
Cause-specific survival according to age and stage of patients with early aggressive NHL.

. The CSS at 10 years for the patients aged 60 years and over was 47%, compared to 75% for the younger group. Combining age and stage information divides the cohort into groups with widely divergent outcomes; CSS at 10 years in young stage I patients was 79%, compared to only 31% in older patients with stage II disease (Figure 5

). The importance of advanced age as a risk factor is emphasised by the inferior long-term survival of older patients with stage I disease even compared to those with more advanced (stage II) disease in the younger group.

## DISCUSSION

The overall CR rate of 78%, and 10 year DFS, OS and CSS rates of 44, 51 and 63%, respectively, are comparable with those achieved by other groups treating early NHL with RT alone (Timothy *et al*, 1980; Hagberg *et al*, 1989). Failure to achieve CR was generally due to the appearance of disease elsewhere, not detected at staging, within 3 months of treatment, rather than a result of radiation resistance. Multivariate analysis reveals groups of patients with significantly poorer prognosis. Increased age and stage are recognised as adverse components of the IPI in NHL (International Non-Hodgkin's Lymphoma Prognostic Factors Project, 1993), and old age was included in the modified IPI applied to early-stage NHL (Miller *et al*, 1998). Elderly and younger patients in this BNLI series enjoyed similar response rates following treatment with RT alone; however, in the elderly survival was significantly inferior. CSS at 10 years was 47% in patients older than 60 years compared with 75% for younger patients. 10-year DFS was similarly poor in the elderly (35% for patients older than 60 years and 52% for those younger). The similarity between DFS and CSS in this group is indicative of the failure of salvage therapy to produce meaningful survival benefit, although details of salvage treatment are not available.

This analysis includes 38 patients treated before 1980 and 255 treated before 1990. Disease-free survival, but not OS or CSS, was significantly superior in patients treated more recently (not shown). However, differences between DFS, OS and CSS for the two age groups remained highly significant when only those patients treated since 1990 were analysed (*P*=0.004, <0.001 and <0.001, respectively). This suggests that the inferior results for older patients reported here do not merely reflect obsolete standards of diagnosis, treatment or supportive care.

In addition to age and stage, multivariate analysis found ESR to be an adverse predictor of DFS. Like LDH, which was not recorded in the earlier years of this series, ESR is likely to serve as a surrogate for disease activity. Similarly, albumin is an independent predictor of CSS. Like ESR, albumin may correlate with disease activity, but may also integrate a measure of comorbidity, which was not itself recorded but is likely to be a predictor of poor outcome in older patients.

The poor outcome in elderly patients treated with RT alone suggests that this group should also be offered systemic therapy. The perception that the elderly respond to and tolerate chemotherapy poorly is not necessarily supported by the evidence (Jones *et al*, 1989), and short-course chemotherapy was originally specifically designed for the elderly (O'Reilly *et al*, 1990). Algorithms have been developed that allow the identification of elderly patients who are more or less functionally impaired by the ageing process (Saliba *et al*, 2001). This method may be used to select patients for treatment according to their true fitness rather than by chronological age alone. Nevertheless, ageing is associated with reduced functional reserve in many organ systems including bone marrow (Chatta and Dale, 1996). This has been perceived as a major barrier to systemic chemotherapy in the elderly because myelosuppression is the most frequent dose-limiting toxicity of such treatment. However, recombinant growth factors are now widely available and are effective in many tumour types including aggressive NHL (Pettengell *et al*, 1992; Zinzani *et al*, 1997). The benefits of growth factors extend to the elderly despite their depleted reserve (Balducci and Lyman, 2001).

A recent comparison of short-course CHOP chemotherapy with or without involved field RT in elderly patients with localised disease showed equivalent overall and CSS in the two groups (Fillet *et al*, 2002). This implies that the addition of RT to short-course chemotherapy provides no additional benefit. Indeed, the addition of RT may be detrimental in the oldest patients as subgroup analysis showed superior OS following chemotherapy alone in those over 69 years (Fillet *et al*, 2002).

The poor outcome reported here for older patients treated with RT alone, even when selected for early stage, indicates that chemotherapy should not be omitted in the elderly for the reason that their age in itself predicts poor outcome. Thus fit older patients with early-stage lymphoma should be offered CMT or even chemotherapy alone.
